# Bibliometric analysis of European publications between 2001 and 2016 on concentrations of selected elements in mushrooms

**DOI:** 10.1007/s11356-020-08693-5

**Published:** 2020-04-23

**Authors:** Paweł Świsłowski, Agnieszka Dołhańczuk-Śródka, Małgorzata Rajfur

**Affiliations:** Institute of Environmental Engineering and Biotechnology, University of Opole, B. Kominka 6a Street, 45-032 Opole, Poland

**Keywords:** Mushrooms, Heavy metals, Review, Bibliometric analysis

## Abstract

This article presents a bibliometric study of 200 European publications released between 2001 and 2016, about the contamination of mushrooms by selected elements. The analysis includes figures on the type of analyte, its concentration, the species of fungi, and its country of origin. In the literature review, 492 species of mushrooms (wild-growing and cultured) found in 26 European countries and their concentration of 74 associated elements were analysed. The papers, which dealt mainly with the heavy metal (Cd, Cu, Fe, Pb, and Zn) concentrations of mushrooms, primarily came from Turkey, Poland, Spain, and the Czech Republic. More than 50% of the publications provided data about edible mushrooms. The results of the bibliometric analysis showed that over the 16 years, European research on fungal contamination by selected analytes has not lessened in popularity and is ongoing. Many of the studies underlined the need to assess the risk to human health arising from the consumption of contaminated mushrooms taken from various habitats. These results were the effect of, among other things, the strong interest in studies carried out on edible species, in which concentrations of mainly heavy metals that are dangerous to health and are marked were indicated (Cd, Pb, and Hg).

**Electronic supplementary material:**

The online version of this article (10.1007/s11356-020-08693-5) contains supplementary material, which is available to authorized users.

## Introduction

Due to their organoleptic (taste) characteristics, mushrooms are a valuable product that are used in a range of dishes. The dry matter content is very low, usually around 100 g/kg. The low proportion of protein, fat, and carbohydrates result in a low nutritional and energy value. The potassium and phosphorus content of the fungi is higher than in most vegetables. The mushroom concentrates nutrients and minerals from the soil through the hyphae; however, they are not actively taken in from the air (Kalač 2009; Falandysz 2017). Mushrooms contain microelements that are indispensable for the functioning of the human organism, but they can also concentrate heavy metals such as cadmium, mercury, and lead (Demirbaş 2001b). The fruiting bodies of edible mushrooms might contain high concentrations of macro- and micro-elements. The ability to take up high amounts of trace elements results from the specific structure of the mycelium: the uncovered surface of the vegetative cells and the hyphae’s large surface area (Işiloǧlu et al. 2001a). Generally, in the fruiting body of the mushrooms, heavy metals are stored bound to proteins, especially to low molecular weight ones (Cuny et al. 2001; Demirbaş 2001a). It was evidenced that the uptake of metals from soil is a species characteristic and the level of concentration of individual microelements in the fungi is genetically conditioned (Işiloǧlu et al. 2001b).

The papers included in this bibliometric analysis (literature reviews in the database) are concerned with the quantification of some important elements present in the fruiting body of some mushrooms; therefore, they contain data only about the concentration ranges of these elements. This article presents a new approach to the presentation and analysis of data on concentrations of selected elements in mushrooms. The abovementioned publications were categorised according to their year of issue, the author’s country of origin, and the most frequently studied species of fungi. Furthermore, the article also includes the values of the highest concentrations of the most frequently studied heavy metals found in fungi. Additionally, a list of fungi species has been presented, together with their current, relevant scientific name (colloquial names, synonyms, or outdated names appear in many publications). The publications whose authors assessed the health risks arising from the consumption of contaminated mushrooms (i.e. with heavy/toxic metals) are also listed according to the different indices (Falandysz and Drewnowska 2015; Melgar et al. 2014; Zsigmond et al. 2015).

This article contains a bibliometric analysis of 200 European publications about the concentrations of selected elements in mushrooms that were published between 2001 and 2016. We evaluated these articles relying on some important aspects: the edibility of mushrooms (edible and non-edible/poisonous species), the most studied elements (type, incidence, and concentration in the fruiting body), and health risks related to consumption (contamination level of mushrooms with heavy metals).

## Materials and methods

The articles considered in this bibliometric survey were selected from various online databases such as ScienceDirect, Springer, Scopus, and Web of Science. These databases are the main sources for monitoring the progress of scientific research. A total of 200 articles from the years 2001–2016 were analysed. Publications from consecutive years do not represent all the published material; however, an attempt was made to select the most ‘popular’ (article citation index) articles in a given year. We tried to collect at least ten publications from each year. Table 1 presents a list of authors covered in the literature survey. Because authors occasionally provided synonyms for the names of the same species, as well as sometimes using outdated phrases from mushroom nomenclature, we have used the current definitions from the *Catalogue of Life: 2019 Annual Checklist and Index Fungorum*. For example, due to the conflicting description regarding the edibility of the species *Tricholoma fracticum* (the mushroom is described as edible in one publication and non-edible in another), we decided to define the edibility of a species using the United Nations FAO (Boa 2004), the MycoKey 4.1 program (MK), and the following selected Internet pages: Wikipedia (W), Fungipedia (F), www.wildfooduk.com (UK), and www.mycodb.fr (MDB). The Supplementary data section contains all the numerical data concerning elements, mushroom species, and the countries present in the list. Our main task was to search for a relevant literature entry (year, location of research) and select the data of interest (element, mushroom species studied). The article was based on reviews, which were the inspiration for this work (Kalač 2010; Kalač and Svoboda 2000). These publications were most frequently based on the analyses of concentrations of individual elements in mushrooms (Mogîldea 2016; Kalač et al. 2004; Román et al. 2006), which were related to publications on the biomonitoring of certain areas for heavy metal pollution in selected mushroom species, e.g. Świsłowski and Rajfur (2018). This article is based on a systematic literature review, following the example of other bibliometric papers (Chang and Ho 2010; de Freitas and Alves-Souza 2019).

**Table 1:** List of authors of works from the period 2001 to 2016

Year	Authors	Number of countries	Number of journals
2001	Blanuša et al. 2001; Cuny et al. 2001; Demirbaş 2001a, b; Işiloǧlu et al. 2001a; b; Falandysz et al. 2001a; b; Falandysz and Bielawski 2001; Marzano et al. 2001; Mattila and Ko 2001; Zimmermannová et al. 2001	Croatia, 1 Finland, 1 France, 1 Italy, 1 Poland, 2 Slovakia, 1 Sweden, 1 Turkey, 4	*Archives of Environmental Contamination and Toxicology*, 2 *Ekologia (Bratislava)*, 1 *Environmental Research*, 1 *Food Additives & Contaminants*, 1 *Food Chemistry*, 3 *Journal of Agricultural and Food Chemistry*, *1* *Journal of AOAC International*, 1 *Polish Journal of Environmental Studies*, 1 *Water, Air, and Soil Pollution*, 1
2002	Baldrian and Gabriel 2002; Demirbaş 2002; Dernovics et al. 2002; Falandysz et al. 2002a; b; Larsen et al. 2002; Lodenius et al. 2002; Mietelski et al. 2002; Ott et al. 2002; Sivrikaya et al. 2002; Svoboda et al. 2002; Collin-Hansen et al. 2002	Czech Republic, 2 Denmark, 1 Finland, 1 Germany, 1 Hungary, 1 Norway, 1 Poland, 2 Ukraine and Spain, 1 Turkey, 2	*Analytical and Bioanalytical Chemistry*, 1 *Applied Radiation and Isotopes*, 1 *Archives of Environmental Contamination and Toxicology*, 1 *Bulletin of Environmental Contamination and Toxicology*, 1 *Environment International*, 1 *FEMS Microbiology Ecology*, 1 *FEMS Microbiology Letters*, 1 *Food Additives and Contaminants*, 1 *Food Chemistry*, 3 *Geochemistry Exploration Environment Analysis*, 1
2003	Adriaensen et al. 2003; Alonso et al. 2003; Collin-Hansen et al. 2003; Djingova et al. 2003; Falandysz et al. 2003a; b; Perkiömäki et al. 2003; Hatvani and Mécs 2003; Svoboda and Kalač 2003; Tüzen 2003; Vetter 2003a, b; Yilmaz et al. 2003	Belgium, 1 Czech Republic, 1 Finland, 1 Germany, 1 Hungary, 3 Norway, 1 Poland, 2 Spain, 1 Turkey, 2	*Archives of Environmental Contamination and Toxicology*, 1 *Bulletin of Environmental Contamination and Toxicology*, 1 *Canadian Journal of Forest Research*, 1 *Ecotoxicology and Environmental Safety*, 1 *European Food Research and Technology*, 1 *Food Chemistry*, 2 *Journal de Physique IV France*, 1 *Microchemical Journal*, 1 *New Phytologist*, 1 *The Science of the Total Environment*, 1 *Turkish Journal of Botany*, 1 *Water, Air, and Soil Pollution*, 1
2004	Colpaert et al. 2004; Isildak et al. 2004; Krupa and Kozdrój 2004; Malinowska et al. 2004; Mendil et al. 2004; Moreno-Rojas et al. 2004; Muller et al. 2004; Nikkarinen and Mertanen 2004; Řanda and Kučera 2004; Turkekul et al. 2004; Vetter 2004; Yeşil et al. 2004	Belgium, 2 Czech Republic, 1 Finland, 1 Hungary, 1 Poland, 2 Spain, 1 Turkey, 4	*Bulletin of Environmental Contamination and Toxicology*, 1 *European Food Research and Technology*, 1 *Food Chemistry*, 5 *Journal of Food Composition and Analysis*, 1 *Journal of Radioanalytical and Nuclear Chemistry*, 1 *New Phytologist*, 2 *World Journal of Microbiology & Biotechnology*, 1
2005	Borovička et al. 2005; Carvalho et al. 2005; Collin-Hansen et al. 2005a, b; Díaz Huerta et al. 2005; Fomina et al. 2005; García et al. 2005; Mendil et al. 2005; Rudawska and Leski 2005a; b; Soeroes et al. 2005; Soylak et al. 2005; Tüzen and Soylak 2005; Vetter 2005a, b; Vetter and Berta 2005	Belgium, Ireland, and Great Britain, 1 Czech Republic and Slovakia, 1 Hungary, 4 Norway, 2 Poland, 2 Portugal, 1 Spain, 2 Turkey, 3	*Analytica Chimica Acta*, 1 *Analytical Sciences*, 1 *Bulletin of Environmental Contamination and Toxicology*, 1 *Food Chemistry*, 4 *Food Control*, 2 *Journal of Chemical Technology and Biotechnology*, 1 *Mycologia*, 1 *Mycological Research*, 3 *Science of the Total Environment*, 1 *Soil Biology & Biochemistry*, 1
2006	Benbrahim et al. 2006; Borovička et al. 2006; Cocchi et al. 2006; Sesli and Dalman 2006; Konuk et al. 2006; Malinowska et al. 2006; Sesli and Tuzen 2006; Moilanen et al. 2006; Sesli 2006; Svoboda et al. 2006; Weeks et al. 2006	Czech Republic, 1 Czech Republic and Slovakia, 1 Finland, 1 France, 1 Great Britain, 1 Italy, 1 Poland, 1 Turkey, 4	*Asian Journal of Chemistry*, 2 *Chemosphere*, 1 *Environmental Pollution*, 1 *Food Additives & Contaminants*, 1 *Food Chemistry*, 3 *Forest Ecology and Management*, 1 *Fresenius Environmental Bulletin*, 1 *Pakistan Journal of Botany*, 1
2007	Borovička and Řanda 2007; Borovička et al. 2007; Falandysz and Bielawski 2007; Falandysz et al. 2007; Figueiredo et al. 2007; Isildak et al. 2007; Komárek et al. 2007; Melgar et al. 2007; Nováčková et al. 2007; Omil et al. 2007; Ouzouni et al. 2007; Tüzen et al. 2007; Yamaç et al. 2007	Czech Republic, 3 Czech Republic and Slovakia, 1 Greece, 1 Poland, 2 Portugal, 1 Spain, 2 Turkey, 3	*Analytical Letters*, 1 *Ekologia (Bratislava)*, 1 *Environment International*, 1 *Food Chemistry*, 2 *Food Control*, 1 *Journal of Agricultural and Food Chemistry*, 1 *Journal of Environmental Science and Health, Part A*, 1 *Journal of Food Composition and Analysis*, 1 *Mycological Progress*, 1 *Mycological Research*, 1 *Science of the Total Environment*, 2
2008	Chudzyński and Falandysz 2008; Ertugay and Bayhan 2008; Falandysz and Gucia 2008; Falandysz et al. 2008; Johansson et al. 2008; Sesli et al. 2008; Svoboda and Chrastný 2008; Tasdemir et al. 2008; Yaǧiz et al. 2008; Žunić et al. 2008	Czech Republic, 1 Poland, 3 Serbia, 1 Sweden, 1 Turkey, 4	*Chemosphere*, 1 *Environmental Geochemistry and Health*, 1 *Food Additives & Contaminants: Part A*, 1 *Fresenius Environmental Bulletin*, 1 *Journal of Environmental Radioactivity*, 1 *Journal of Environmental Science and Health, Part A*, 1 *Journal of Hazardous Materials*, 2 *Journal of Microbiology and Biotechnology*, 1 *Soil Biology & Biochemistry*, 1
2009	Brzostowski et al. 2009; Campos et al. 2009; Chudzyński et al. 2009; Duran et al. 2009; García et al. 2009; Gençcelep et al. 2009; Gonzálvez et al. 2009; Guillén et al. 2009; Gursoy et al. 2009; Melgar et al. 2009; Ouzouni et al. 2009; Krpata et al. 2009; Gorbunova et al. 2009; Elekes et al. 2009	Austria, 1 Greece, 1 Poland, 2 Romania, 1 Russia, 1 Spain, 5 Turkey, 3	*Annals. Food Science and Technology*, 1 *Biometals*, 1 *Bulletin of Environmental Contamination and Toxicology*, 1 *Chemical Analysis*, 1 *Contemporary Problems of Ecology*, 1 *Environmental Pollution*, 1 *Food and Chemical Toxicology*, 1 *Food Chemistry*, 3 *Journal of Hazardous Materials*, 2 *Science of the Total Environment*, 2
2010	Borovička et al. 2010a; b; Çayir et al. 2010; Ertugay and Bayhan 2010; Frankowska et al. 2010; Karadeniz and Yaprak 2010; Ozturk et al. 2010; Radulescu et al. 2010a; b; c; Sarikurkcu et al. 2010; Zhang et al. 2010	Czech Republic, 2 Poland, 2 Romania, 3 Turkey, 5	*African Journal of Agricultural Research*, 1 *Biological Trace Element Research*, 1 *Bulletin of Environmental Contamination and Toxicology*, 1 *Desalination*, 1 *Food Additives & Contaminants: Part B*, 1 *Food and Chemical Toxicology*, 1 *Journal of Consumer Protection and Food Safety*, 1 *Journal of Radioanalytical and Nuclear Chemistry*, 1 *Ovidius University Annals of Chemistry*, 1 *Romanian Biotechnological Letters*, 1 *Science of the Total Environment*, 1 *Soil Biology & Biochemistry*, 1
2011	Ayaz et al. 2011; Borovička et al. 2011; Brzostowski et al. 2011; Busuioc et al. 2011; Campos and Tejera 2011; Chudzyński et al. 2011; Costa-Silva et al. 2011; Kula et al. 2011; Rieder et al. 2011; Osobová et al. 2011; Sarikurkcu et al. 2011; Stihi et al. 2011	Czech Republic, 2 Poland, 2 Portugal, 1 Romania, 2 Spain, 1 Switzerland, 1 Turkey, 3	*Biological Trace Element Research*, 1 *Biometals*, 1 *Bulletin of Environmental Contamination and Toxicology*, 1 *Bulletin UASVM Agriculture*, 1 *Environmental Pollution*, 1 *Environmental Science and Pollution Research*, 1 *Food and Nutrition Sciences*, 1 *Food Chemistry*, 3 *Journal of Environmental Science and Health, Part A*, 1 *New Phytologist*, 1
2012	Aloupi et al. 2012; Cremades et al. 2012; Giannaccini et al. 2012; Gryndler et al. 2012; Gucia et al. 2012; Maćkiewicz and Falandysz 2012; Milinkovic et al. 2012; Mititelu et al. 2012; Sarikurkcu et al. 2012; Şen et al. 2012; Škrbić et al. 2012; Vinichuk 2012	Czech Republic, 1 Greece, 1 Italy, 1 Poland, 2 Romania, 1 Serbia, 2 Spain, 1 Sweden, 1 Turkey, 2	*Biological Trace Element Research*, 1 *Biometals*, 1 *Bulletin of Environmental Contamination and Toxicology*, 1 *Ecological Indicators*, 1 *Ecology of Food and Nutrition*, 1 *Ecotoxicology and Environmental Safety*, 1 *Environmental Monitoring and Assessment*, 1 *Environmental Science and Pollution Research*, 1 *Food Chemistry*, 1 *ISRN Ecology*, 1 *Journal of Environmental Protection and Ecology*, 1 *Proceedings of 6th Central European Congress on Food*, 1
2013	Daillant et al. 2013; García-Delgado et al. 2013; García et al. 2013; Gramss and Voigt 2013; Gwynn et al. 2013; Miklavčič et al. 2013; Mirończuk-Chodakowska et al. 2013; Özcan et al. 2013; Petkovšek and Pokorny 2013; Ruytinx et al. 2013; Severoglu et al. 2013; Slávik et al. 2013; Zhang et al. 2013	Belgium, 1 France, 1 Germany, 1 Norway, 1 Poland, 2 Slovakia, 1 Slovenia, 2 Spain, 2 Turkey, 2	*Bulletin of Environmental Contamination and Toxicology*, 1 *Environmental Monitoring and Assessment*, 1 *Environmental Research*, 1 *Environmental Science and Pollution Research*, 1 *Food and Chemical Toxicology*, 1 *International Journal of Environmental Science and Technology*, 1 *Journal of Environmental Radioactivity*, 1 *Journal of Microbiology, Biotechnology and Food Sciences*, 1 *Journal of Mountain Science*, 1 *Journal of Radioanalytical and Nuclear Chemistry*, 1 *Metallomics*, 1 *Polish Journal of Environmental Studies*, 1 *Science of the Total Environment*, 1
2014	Baumann et al. 2014; Borovička et al. 2014; Drewnowska et al. 2014; Dryżałowska and Falandysz 2014; Gezer and Kaygusuz 2014; Kubrová et al. 2014; Llorente-Mirandes et al. 2014; Melgar et al. 2014; Nagy et al. 2014; Rakić et al. 2014; Sácký et al. 2014; Širić et al. 2014	Croatia, 1 Czech Republic, 3 Germany, 1 Poland, 2 Romania, 1 Serbia, 1 Spain, 2 Turkey, 1	*Applied Geochemistry*, 1 *Ecotoxicology and Environmental Safety*, 1 *Environmental Progress & Sustainable Energy*, 1 *Environmental Science and Pollution Research*, 2 *Food and Chemical Toxicology*, 1 *Food Chemistry*, 1 *Fungal Genetics and Biology*, 1 *Journal of Environmental Protection and Ecology*, 1 *Journal of Environmental Science and Health, Part B*, 1 *Journal of Hazardous Materials*, 1 *Periodicum Biologorum*, 1
2015	Cordeiro et al. 2015; Dementyev et al. 2015a, b; García-Delgado et al. 2015; Falandysz and Drewnowska 2015; García et al. 2015; Koroleva and Okhrimenko 2015; Krasińska and Falandysz 2015; Ostos et al. 2015; Sarikurkcu et al. 2015; Schlecht and Säumel 2015; Vaario et al. 2015; Zsigmond et al. 2015	Finland, 1 Germany, 1 Poland, 2 Romania, 1 Russia, 3 Spain, 4 Turkey, 1	*Acta Universitatis Sapientiae, Agriculture and Environment*, 1 *Doklady Biochemistry and Biophysics*, 1 *Ecotoxicology and Environmental Safety*, 2 *Environmental Pollution*, 1 *Food Additives & Contaminants: Part A*, 1 *Food Chemistry*, 1 *Journal of Environmental Science and Health, Part A*, 1 *Journal of Food Composition and Analysis*, 1 *Journal of Hazardous Materials*, 1 *Mycorrhiza*, 1 *Pollution Atmosphérique*, 1 *Radiochemistry*, 1
2016	Cejpková et al. 2016; Dimitrijevic et al. 2016; Frutos et al. 2016; Gabriel et al. 2016; Kojta and Falandysz 2016; Kosanić et al. 2016; Krasińska and Falandysz 2016; Llorente-Mirandes et al. 2016; Melgar et al. 2016; Širić et al. 2016a; b; Stefanović et al. 2016a; b	Croatia, 2 Czech Republic, 2 Poland, 2 Serbia, 4 Spain, 3	*Ecotoxicology and Environmental Safety*, 1 *Environmental Earth Sciences*, 1 *Environmental Pollution*, 1 *Environmental Science and Pollution Research*, 4 *European Food Research and Technology*, 1 *Food and Chemical Toxicology*, 1 *Food Chemistry*, 1 *International Journal of Environmental Science and Technology*, 1 *Journal of Food and Drug Analysis*, 1 *Šumarski list*, 1

## Results and discussion

The average number of publications per year was 13. Table 2 contains a list of the ten most frequently mentioned mushroom species in the above publications.

**Table 2:** Most frequently mentioned mushroom species in the publications

Species	Number	Percentage of publications (%)
*Boletus edulis*	57	28.5
*Macrolepiota procera*	49	24.5
*Cantharellus cibarius*	44	22.0
*Imleria badia*		
*Lactarius deliciosus*	39	19.5
*Lepista nuda*	35	17.5
*Leccinum scabrum*	34	17.0
*Xerocomellus chrysenteron* (Bull.) Šutara, 2008*	32	16.0
*Agaricus bisporus*	31	15.5
*Agaricus campestris*		

*The name *Xerocomellus chrysenteron* is already outdated

The *Boletus edulis* was the most frequently studied species, and it was present in 57 articles out of 200. The articles dealt with a total number of 492 species of edible (262) and non-edible mushrooms (226). The list included four mushroom species (*Helvella leptopodra*, *Hypholoma pudorinus*, *Russula nigrescens*, *Suillus elegant*) that were only mentioned in these publications and have not been identified elsewhere. This could have been caused by an erroneously quoted name or the use of a synonym of the approved name. In Table 2, only the edible species from the general list are shown. This confirms that European researchers focused on edible mushroom species (more than half of the 492 fungi species included in the analysed articles). The phrase ‘non-edible’ mushrooms signifies those that have no culinary value, those whose consumption can be hazardous to health, and poisonous ones. The species *Amanita muscaria* was the most frequently mentioned non-edible mushroom species (26 times).

Table 3 contains five of the most frequently occurring elements out of the 74 mentioned. The Supplementary data section contains both alkali metals, non-metals, radioisotopes, and heavy metals. The last group occurred most frequently in the list.

**Table 3:** The number of occurrences of individual elements in the articles

Chemical element	Number	Species with the highest concentration of the element (mg kg^-1^ dry mass)	Habitat, where the mushrooms were collected	References
Cd	108	*Paxillus involutus* (Batsch) Fr., 1838 (3964 ± 611)	Laboratory – grown in a liquid medium	Ott et al. 2002
Zn	101	*Lepista nuda* (Bull.) Cooke, 1871 (4325 ± 298)	pine forest site, Çınardibi, Turkey	Karadeniz and Yaprak 2010
Pb	98	*Amanita citrina* Pers., 1797 (895 ± 41)	Industrial desert surrounding a nonferrous (zinc and lead) works in Miasteczko Slaskie, Poland	Krupa and Kozdrój 2004
Cu	96	*Xerocomellus chrysenteron* (Bull.) Šutara, 2008 (502)	Four sites in a rural area, unpolluted region near the town of Moravský Krumlov in south-western Moravia, Czech Republic	Svoboda and Chrastný 2008
Fe	76	*Lycoperdon perlatum* Pers., 1796 (24,600 ± 368)	The province of Mugla in the South-Aegean Region of Turkey	Sarikurkcu et al. 2015

The majority of publications contained studies on the mushrooms’ capacity for concentrating elements (e.g. migration mechanisms), or their possible harmfulness (polluted with heavy metals) when consumed by humans. The analyses of Cd, Pb, and Zn concentrations and their presence in mushrooms were due to the fact that many authors indicated the health-related aspects of consuming mushrooms contaminated by heavy metals (Table 4).

**Table 4:** List of indices

Indices	References
Hazard index (HI)	Falandysz et al. 2002a; Falandysz et al. 2003a; Falandysz et al. 2003b; Zsigmond et al. 2015
Hazard quotient (HQ)	Zsigmond et al. 2015
Provisional tolerable weekly intake (PTWI)	Blanuša et al. 2001;Çayir et al. 2010; Chudzyński et al. 2009; Chudzyński et al. 2011; Dimitrijevic et al. 2016; Drewnowska et al. 2014; Dryżałowska and Falandysz 2014; Falandysz and Drewnowska 2015; Frankowska et al. 2010; García et al. 2009; Giannaccini et al. 2012; Gucia et al. 2012; Gursoy et al. 2009; Komárek et al. 2007; Krasińska and Falandysz 2015; Krasińska and Falandysz 2016; Larsen et al. 2002; Maćkiewicz and Falandysz 2012; Malinowska et al. 2004; Miklavčič et al. 2013; Mirończuk-Chodakowska et al. 2013; Ostos et al. 2015; Ouzouni et al. 2007; Petkovšek and Pokorny 2013; Rudawska and Leski 2005a; Sarikurkcu et al. 2011; Sarikurkcu et al. 2012; Stefanović et al. 2016b; Svoboda et al. 2006
Reference dose (RfD)	Chudzyński et al. 2009; Chudzyński et al. 2011; Drewnowska et al. 2014; Dryżałowska and Falandysz 2014; Falandysz and Drewnowska 2015; Frankowska et al. 2010; Krasińska and Falandysz 2016; Maćkiewicz and Falandysz 2012; Melgar et al. 2014; Stefanović et al. 2016a; Zsigmond et al. 2015
Tolerable weekly intake (TWI)	Dimitrijevic et al. 2016; Gucia et al. 2012; Melgar et al. 2016; Ozturk et al. 2010; Schlecht and Säumel 2015; Stefanović et al. 2016b
Acceptable daily intake (ADI)	Alonso et al. 2003
Dietary reference intake (DRI)	Cocchi et al. 2006; Costa-Silva et al. 2011
Provisional tolerable daily intake (PTDI)	Sesli et al. 2008
Recommended dietary allowance (RDA)	Busuioc et al. 2011; Çayir et al. 2010; García et al. 2013; Stefanović et al. 2016a
Recommended daily intake (RDI)	Aloupi et al. 2012; Dimitrijevic et al. 2016
Tolerable daily intake (TDI)	Aloupi et al. 2012; Stefanović et al. 2016b
Probable daily intake (PDI)	Miklavčič et al. 2013

The most frequently discussed element was cadmium (Table 3). The influence of different concentrations of this element on growth and its content in *Paxillus involutus* was determined by the highest concentration of Cd (3964 ± 611 mg kg^-1^ dry mass). This mushroom was isolated from its fruiting bodies at a mining site contaminated with heavy metals, in the Harz mountains, Germany (Ott et al. 2002). However, if we consider the natural cadmium contamination of mushrooms, the highest concentration was found in fungi taken from an area contaminated by a smelter in Lhota near Příbram in the Czech Republic: the concentration of this metal in *I. badia* was 333 mg kg^-1^ d. m. (Cejpková et al. 2016). An equally high concentration (325 mg kg^-1^ d. m.) was recorded in *Agaricus arvensis*, which was collected in Slovenia. The fungi were collected in the vicinity of the largest Slovenian thermal power plant (the Šalek Valley) and near an abandoned lead smelter (the Upper Meža Valley) (Petkovšek and Pokorny 2013). The maximum concentration of Cd permitted by European Union regulation in cultivated *A. bisporus*, *P. ostreatus*, and *L. edodes* is 2.0 mg kg^−1^ d. m., assuming 90% moisture, while for other mushrooms, it is 1.0 mg kg^−1^ fw (10 mg kg^−1^ d. m.) (EU 2008). It should be stressed that mushrooms collected in such areas are unfit for consumption.

The zinc concentrations in mushrooms were analysed in more than half of the publications. Most often Zn concentrations are in the range of 50–150 mg kg^-1^ d. m. However, a higher concentration of Zn was present in *Sarcodon scabrosus*, taken from a pine forest in Turkey (4325 ± 298 mg kg^-1^ d. m.). This result differed significantly from the abovementioned average concentrations presented in the literature. The concentration of Zn in fungi seems to be similar to, or higher than, that found in the soil. The only species known to accumulate Zn in a significant way is *Russula atropurpurea*. This species, taken from unpolluted areas in the Czech Republic and Slovakia, contained as high Zn as 1062 mg kg^-1^ d. m. (Borovička and Řanda 2007). The high concentrations of zinc in sporocarps of this species are related to the presence of functional peptides, which bind this metal (Leonhardt et al. 2014).

The habitat from which fungi are taken has a direct impact on the levels of contamination of mushrooms by selected elements (including heavy metals). This was also the case of *Amanita citrina*, taken from industrialised areas in Upper Silesia in Poland. The concentration of lead in the fungus was 895 mg kg^-1^ d. m. The influence of zinc and lead smelters, which have been emitting pollutants in this area since the end of the nineteenth century, is undeniable (Krupa and Kozdrój 2004). Lead concentration in *Macrolepiota procera* was found to be of 171 mg kg^-1^ d. m. The habitat of the fungi was contaminated with Pb due to former metallurgical and mining activities (Petkovšek and Pokorny 2013). The low mobility of Pb and its ensuing rapid accumulation in mushroom stem are the main reasons why Pb is found chiefly in stems rather than mushroom caps (Komárek et al. 2007). An example is the 2007 study carried out near a Pb smelter in the highly polluted area of Příbram in the Czech Republic. In the upper soil layer of this area, lead was measured at a concentration of 36,234 mg kg^-1^, while the concentrations in the stipe of *B. edulis* growing in this study area was found to be 165 mg kg^-1^ d. m. (Komárek et al. 2007).

The copper content of mushrooms is usually 100–300 mg kg^-1^ d. m. During the period under consideration in the analysed European literature, a very high concentration of this analyte was determined in fungi collected from unpolluted areas of the Czech Republic, which makes this level of contamination puzzling: see Table 3 (Svoboda and Chrastný 2008). A copper concentration of 427 mg kg^-1^ d. m. was measured in *B. edulis* in Norway, where mushrooms were collected from around a copper smelter (Collin-Hansen et al. 2002). In *Agaricus xanthodermus*, taken from areas with geochemical characteristics determined as polymetallic ores Pb-Cu-Zn-Ag, the concentration of copper was 420 ± 14 mg kg^-1^ d. m. (Řanda and Kučera 2004). Taking fungi from such areas means that the Cu content may be elevated (copper concentration is usually 100–300 mg kg^-1^ d. m.), taking into account the contamination of a given site (Kalač and Svoboda 2000; Kalač 2010).

The highest concentrations of iron were found in mushrooms collected in Turkey (Table 3). The high concentrations of this metal can be attributed to the industrial activity in this region. In *Lepista nuda*, which was collected from Türkmenbaba Mountain in the Eskişehir forest area, an Fe concentration of 11,460 ± 6 mg kg^-1^ d. m. was measured. The results presented in this publication, for this species of fungus, were also very high for other elements (Pb, Mn, and Cu) and differed from the concentrations found in other samples of mushrooms collected from Turkey (Yamaç et al. 2007). In turn, in *Omphalotus olearius*, taken from the forest along the Balıkesir-Manisa highway, 9685 mg kg^-1^ dry weight of iron was found. These areas were exposed to traffic pollution for many years (Yilmaz et al. 2003).

On the basis of the literature research we carried out, it can be concluded that high concentrations of heavy metals depend on the genetic properties of a given species (the ability of individual species to accumulate analytes (hyperaccumulation) (Falandysz and Borovička 2013; Sácký et al. 2016), and on the level of contamination in the habitat from which the material for testing was sampled (Mleczek et al. 2016).

Table 4 shows a list of indices that researchers used to assess the health risks associated with the consumption of fungi contaminated by heavy metals. The choice of appropriate indices for analysis depended on the type of test performed, the species of fungus on which the test was performed, and the element whose concentration was determined.

According to the literature analysis, provisional tolerable weekly intake (PTWI) was determined most frequently. The Joint FAO/WHO Expert Committee on Food Additives (JECFA) gives tolerable intake levels for contaminants, expressed on either a daily or a weekly basis. Unlike tolerable daily intake (TDI), the introduction of the term ‘weekly’ is intended to emphasise the need to limit the intake of a substance over a certain period of time, given that many contaminants are not rapidly removed from the body (Herrman and Younes 1999; Türkmen and Budur 2018). In addition, it can be concluded that this parameter was increasingly used in studies over the last decade of the analysis. In the research of many of the authors, they stressed the need to assess the risk to human health arising from the consumption of contaminated mushrooms taken from a given area (Table 4). This was due to the great interest in research carried out on edible species, in which concentrations of, mainly, heavy metals that are dangerous to health (Cd, Pb, and Hg) were determined.

**Fig. 1:**
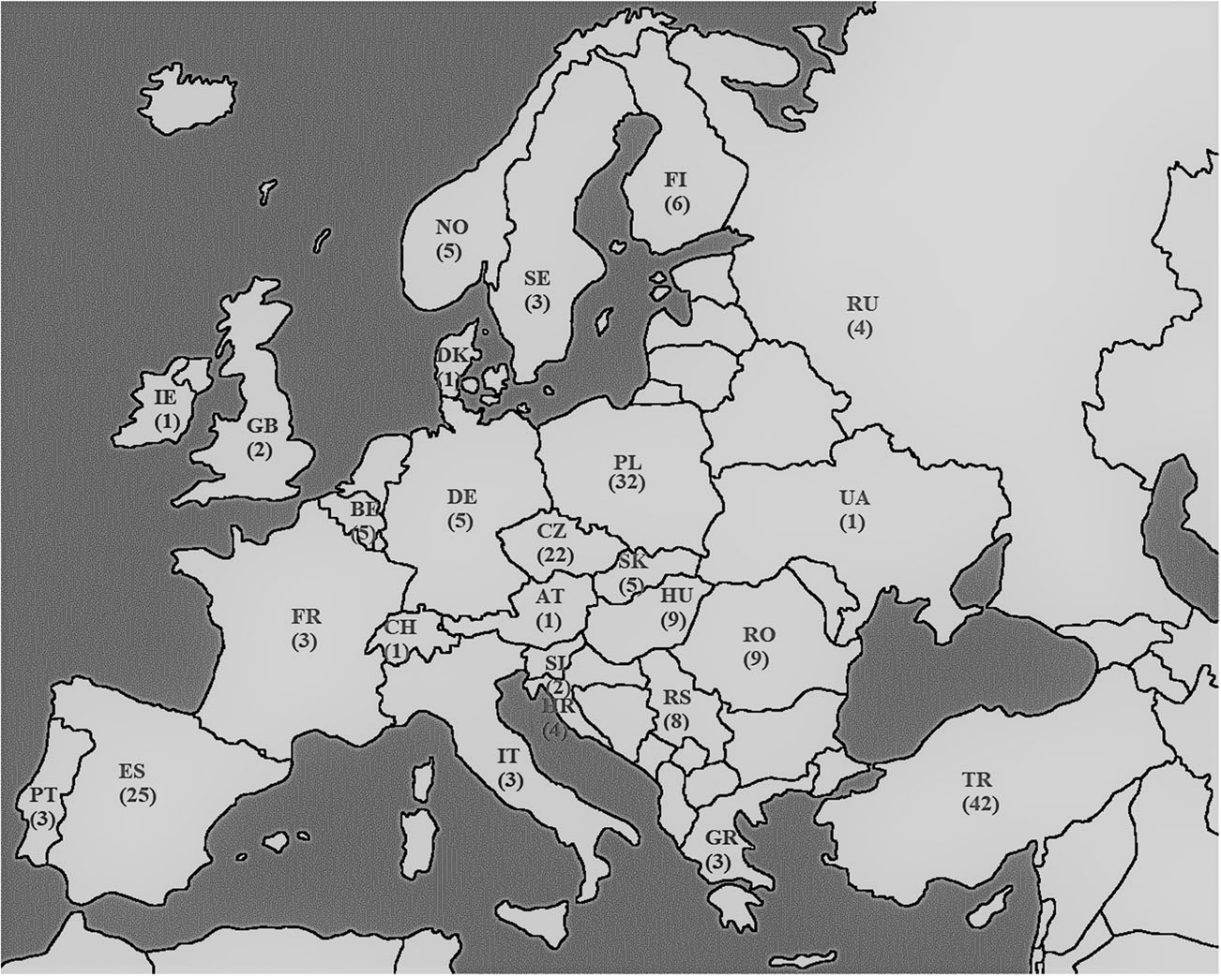
Map of Europe showing the frequency of publications for individual countries

Figure 1 contains a graphical representation of the countries where the discussed publications originated; for each country, the number of studies concerning sample acquisition is provided (for example, mushrooms purchased in shops or wild-grown).

Out of 26 countries, the following four were mentioned most frequently: Turkey (42), Poland (32), Spain (25), and the Czech Republic (22). Many studies about these issues have been carried out in Poland, as was shown earlier (Świsłowski and Rajfur 2017). It should be emphasised that the research teams in these countries are stable and their members produce joint publications. For example, in Poland, these works are produced mainly by Professor J. Falandysz and his teams, such as Falandysz et al. (2017).

## Conclusions

As a result of this bibliometric study of 200 European publications, appearing between 2001 and 2016, on the contamination of mushrooms by selected elements, we concluded that this issue is still popular and relevant. Each year, there is an increase in the number of papers assessing the level of health risks associated with the consumption of fungi contaminated with heavy metals using different indices. The main research being done on the concentration of elements in mushrooms is connected with their heavy metal content and the risks resulting from their consumption. So it is not surprising that more than half of the 492 species of mushrooms appearing in the articles under consideration were edible. These studies were mainly concerned with taking wild fungi from various areas and determining whether selected elements were present in them. There was also no shortage of papers in which different species of mushrooms were cultured and their sorption properties in relation to selected analytes subsequently analysed. The publications also included species of fungi with a natural ability to accumulate elements, thanks to which, the mushrooms can be used for phytoremediation of contaminated soils. The highest number of publications came from Turkey, Poland, Spain, and the Czech Republic; so these countries made the largest contribution to the development of the science of elements in mushrooms and the assessment of the health risks associated with the consumption of contaminated mushrooms.

## Electronic supplementary material

ESM 1 (XLSX 73 kb)
